# Association of Baseline Serum Levels of CXCL5 With the Efficacy of Nivolumab in Advanced Melanoma

**DOI:** 10.3389/fmed.2019.00086

**Published:** 2019-04-26

**Authors:** Taku Fujimura, Yota Sato, Kayo Tanita, Chunbing Lyu, Yumi Kambayashi, Ryo Amagai, Atsushi Otsuka, Yasuhiro Fujisawa, Koji Yoshino, Shigeto Matsushita, Hiroshi Uchi, Yuki Yamamoto, Hiroo Hata, Takeru Funakoshi, Yumi Nonomura, Ryota Tanaka, Hisako Okuhira, Naoko Wada, Akira Hashimoto, Setsuya Aiba

**Affiliations:** ^1^Department of Dermatology, Tohoku University Graduate School of Medicine, Sendai, Japan; ^2^Department of Dermatology, Kyoto University Graduate School of Medicine, Kyoto, Japan; ^3^Department of Dermatology, University of Tsukuba, Tsukuba, Japan; ^4^Department of Dermatology, Tokyo Metropolitan Cancer and Infectious Disease Center Komagome Hospital, Tokyo, Japan; ^5^Department of Dermato-Oncology/Dermatology, National Hospital Organization Kagoshima Medical Center, Kagoshima, Japan; ^6^Department of Dermatology, Kyushu University Graduate School of Medicine, Fukuoka, Japan; ^7^Department of Dermatology, Wakayama Medical University, Wakayama, Japan; ^8^Department of Dermatology, Hokkaido University Graduate School of Medicine, Sapporo, Japan; ^9^Department of Dermatology, Keio University School of Medicine, Tokyo, Japan

**Keywords:** baseline levels of CXCL5, melanoma, nivolumab, prediction of efficacy, nivolumab and ipilimumab combined therapy

## Abstract

Anti-programmed cell death protein 1 (PD1) antibodies are in wide use for the treatment of various cancers. PD1 antibody-based immunotherapy, co-administration of nivolumab and ipilimumab, is one of the optimal immunotherapies, especially in advanced melanoma with high tumor mutation burden. Since this combined therapy leads to a high frequency of serious immune-related adverse events (irAEs) in patients with advanced melanoma, biomarkers are needed to evaluate nivolumab efficacy to avoid serious irAEs caused by ipilimumab. This study analyzed baseline serum levels of CXCL5, CXCL10, and CCL22 in 46 cases of advanced cutaneous melanoma treated with nivolumab. Baseline serum levels of CXCL5 were significantly higher in responders than in non-responders. In contrast, there were no significant differences in baseline serum levels of CXCL10 and CCL22 between responders and non-responders. These results suggest that baseline serum levels of CXCL5 may be useful as a biomarker for identifying patients with advanced cutaneous melanoma most likely to benefit from anti-melanoma immunotherapy.

## Introduction

Anti-programmed cell death protein 1 (PD-1) antibodies such as nivolumab and pembrolizumab are in wide use for the treatment of various cancers, including advanced melanoma (1, 2), but cost-effective analyses of their use are sometimes controversial (3). Therefore, biomarkers for the evaluation of the efficacy of anti-PD1 antibody therapy are needed. Previous clinical studies suggested that the efficacy of nivolumab monotherapy is ~40% in the Caucasian population (2, 4), which contains a high ratio of superficial spreading melanoma (SSM) with high levels of tumor mutation burden (TMB) (5). In contrast, in the Japanese population, there is a high ratio of acral lentiginous melanoma (ALM) and mucosal melanoma (6), which have low levels of TMB (5). The efficacies of nivolumab and pembrolizumab in Japan have been reported to be 34.1% and 24.1%, respectively (7, 8), suggesting that another drug that could enhance the anti-tumor immune response in melanoma is needed.

Ipilimumab is a fully humanized immunoglobulin (Ig)G1 monoclonal antibody that blocks cytotoxic T-lymphocyte antigen (CTLA-4) and is one of the promising drugs that enhance the anti-tumor immune response for patients with advanced melanoma with or without BRAF gene mutation in combination with nivolumab (1, 4, 9). Indeed, the efficacy of this combination therapy for advanced melanoma has been reported to be 57.8% (4), and, therefore, combination therapy with nivolumab and ipilimumab is recommended by the NCCN guideline for cutaneous melanoma as a first-line therapy (10). This combination therapy achieves a high efficacy rate even for the treatment of brain metastases of melanoma (11). In addition, Blank et al. reported that the efficacy of co-administration of nivolumab and ipilimumab does not parallel TMB (12). In addition to co-administration of nivolumab and ipilimumab, sequential administration of nivolumab and ipilimumab with a planned switch leads to high efficacy in the treatment of advanced melanoma (4, 9). On the other hand, both co-administration of nivolumab and ipilimumab and sequential administration of nivolumab and ipilimumab with a planned switch lead to a high frequency of serious immune-related adverse events (irAEs), such as hepatitis, colitis, polyneuropathy, etc., in patients with advanced melanoma (1, 4, 11). Therefore, determining the efficacy of nivolumab monotherapy before starting first-line immune therapy for melanoma is important.

CXCL5 is a chemokine that can recruit not only neutrophils, but also CXCR2+ myeloid-derived suppressor cells (MDSCs) and CXCR2+ monocytes that can be precursors of tumor-associated macrophages (TAMs) (13–15). Indeed, Soler-Cardona et al. reported that CXCL5-overexpressing melanomas had significantly increased lymph node metastases of melanoma (15) caused by the recruitment of immunosuppressive PD-L1-expressing neutrophils, leading to interference with systemic activation of the anti-tumor immune system using poly (I: C) (14). In another report, the recruitment of CXCR2-expressing MDSCs played significant roles in the development of colitis-associated colon cancer (13). These reports suggested the production of CXCL5 in the cancer stroma of melanoma.

In addition to autoimmune-related chemokines, chronic inflammatory chemotactic factors such as CXCL10 are also important for the recruitment of immunosuppressive cells such as regulatory T cells (Tregs) and MDSCs. Jiang et al. reported that, compared to patients with stable disease, advanced melanoma patients had increased levels of IL-1β and CXCL10 in the serum associated with accumulation of monocytic (Mo)-MDSCs and Tregs in peripheral blood, which correlated with the progression-free survival of these patients (16). In addition, other reports also suggested that serum CXCL10 levels were correlated with disease activity in advanced melanomas (17) and angiosarcomas (18). These reports suggested that serum CXCL10 levels may represent disease activity in advanced melanoma.

Not only MDSCs, but Tregs are also important for tumor progression in melanoma (19, 20). Indeed, Johansenn et al. previously reported that Tregs at the tumor sites were correlated with tumor progression in melanoma (20). More recently, Ha et al. reported the significance of high CTLA4 expression for Tregs, leading to selective depletion of Tregs in melanoma, which might be an important tool in designing cancer immunotherapy (21). In addition, as described above, the reduction of CCL22 by TAMs decreases Tregs in the tumor site, which enhances the therapeutic effects of immune therapy in the mouse melanoma model (22). Taken together, these reports suggest that serum CCL22 may be correlated with the efficacy of immune therapy.

From the above findings, in this report, the baseline serum levels of TAM-associated chemokines were investigated in 46 advanced melanoma patients treated with nivolumab.

## Patients and Methods

### Ethics Statement for Human Experiments

The protocol for this human study was approved by the ethics committee of Tohoku University Graduate School of Medicine, Sendai, Japan (Permit No: 2017-1-064). All methods were performed in accordance with the relevant guidelines and regulations. All patients provided their written, informed consent.

### Patients

Data from patients treated with nivolumab were collected from eight institutes in Japan. Patients were eligible if they had unresectable stage III melanoma, or if the patients had stage IV melanoma with accessible cutaneous, subcutaneous, and/or nodal lesions (staging was performed according to the AJCC Staging Manual, 7th edition, 2011). All patients received 2 mg/kg of nivolumab followed by a 3-week rest period or 3 mg/kg of nivolumab followed by 2 weeks of rest, both of which are approved dosing schedules in Japan. Serum was obtained from patients before the administration of nivolumab. The response to nivolumab was assessed according to Response Evaluation Criteria In Solid Tumors.

### Baseline Serum Levels of CXCL5 and CXCL10

Before nivolumab administration, the serum was stored, and serum levels of CXCL5, CXCL10, and CCL22 were then analyzed by enzyme-linked immunoassay (ELISA) according to the protocol provided by the manufacturer (R&D Systems, Minneapolis, MN).

### Statistical Methods

Receiver operating characteristic (ROC) curves were used to calculate cut-off values for serum levels of CXCL5, CXCL10, and CCL22 and areas under the curves (AUCs). Cut-offs were determined using Youden's index 12 (sensitivity + specificity −1) to determine the point of the maximum index value (23). ROC curves were established to evaluate serum levels of CXCL5 and CXCL10 in patients administered nivolumab. All statistical analyses were performed using JMP version 14.1 software (SAS Institute, Tokyo, Japan). For a single comparison of two groups, the Mann-Whitney *U*-test was used. The level of significance was set at *p* < 0.05.

## Results

### Patients

Data were collected from 46 melanoma patients treated with nivolumab (Table 1). The mean patient age was 67 years (range, 33–93 years). Of the patients with melanoma, 58.7% were males, and 41.3% were females. The most common primary tumor site was the extremities (41.3%), followed by mucosal origin (30.4%), trunk (15.2%), head and neck (10.9%), and unknown origin (2.2%).

**Table 1:** Characteristics and serum levels of CXCL5, CXCL10, and CCL22 in patients with cutaneous melanoma.

	**Age (y)**	**Sex**	**Location**	**Efficacy**	**CXCL5 (pg/ml)**	**CXCL10 (pg/ml)**	**CCL22 (pg/ml)**
1	51–60	M	Trunk	SD	226.9	69.31	290.9
2	31–40	F	Extremities	PD	307.7	212.8	814.6
3	61–70	F	Vagina	PD	237.6	117.9	611.2
4	61–70	M	Extremities	PR	497.5	144.4	314.6
5	61–70	M	Extremities	PR	332.6	72.13	401.5
6	81–90	F	Extremities	PR	434.8	355.5	977.3
7	61–70	M	Trunk	PD	862.1	113.0	891.5
8	81–90	F	Extremities	PD	433.9	186.1	1340
9	71–80	M	Head and neck	SD	461.3	97.21	615.7
10	81–90	F	Trunk	PD	314.9	74.89	637.2
11	91–100	M	Extremities	PD	423.4	122.2	582.6
12	71–80	M	Extremities	SD	471.9	84.24	1031
13	61–70	M	Extremities	PD	222.6	202.5	448.7
14	61–70	F	Vagina	SD	667.8	322.7	548.3
15	71–80	M	Trunk	PR	502.8	358.7	603.8
16	71–80	F	Extremities	PR	408.6	550.1	523.0
17	81–90	F	Unknown	SD	940	266.1	701.2
18	71–90	M	Nasal cavity	SD	332.5	188.1	840.2
19	61–70	M	Nasal cavity	PD	162.9	1001	788.0
20	61–70	M	Paranasal	PD	292.1	247.1	678.2
21	61–70	F	Vagina	PD	292.4	368.2	497.1
22	61–70	F	Vagina	PD	271.6	386.9	475.7
23	51–60	F	Conjunctiva	SD	380.9	336.7	355.5
24	81–90	M	Digestive duct	PD	237.4	208.1	438.3
25	61–70	F	Digestive duct	SD	5026	336.8	987.9
26	61–70	F	Trunk	PD	474.3	245.3	84.71
27	71–80	M	Extremities	PD	494.1	116.7	630.4
28	51–60	F	Head and neck	PD	370.5	93.53	983.2
29	31–40	M	Trunk	SD	501.8	97.59	857.8
30	31–40	F	Extremities	PR	407	138.7	963.1
31	71–80	M	Extremities	SD	529.6	108.4	637.7
32	31–40	M	Extremities	PD	687.1	147.1	845.1
33	71–80	F	Extremities	SD	544.7	104.6	935.6
34	71–80	M	Head and neck	PD	701.3	77.79	918.5
35	41–50	M	Extremities	PD	655.4	432.3	617.5
36	71–80	F	Extremities	PR	465.3	184.8	1065
37	61–70	M	Trunk	PR	555.2	105.7	915.5
38	41–50	M	Head and neck	SD	740.9	65.85	830.3
39	41–50	M	Extremities	PD	723.8	54.08	944.6
40	61–70	F	Head and neck	SD	410.7	62.46	470.7
41	71–80	F	Extremities	PR	1196	71.03	606.1
42	61–70	M	Digestive duct	PR	564	190.4	498.5
43	61–70	F	Palate	PR	1600	51.84	619.5
44	51–60	F	Extremities	CR	687.3	80.09	701.3
45	61–70	M	Paranasal	CR	1142	192	211.3
46	61–70	F	Vagina	CR	4939	68.73	573

*Changes of CXCL5, CXCL10, and CCL22 serum levels in each patient (n = 46) before the administration of nivolumab were examined by ELISA. Data for each donor represent the means of duplicate assays*.

*CR, complete response*.

*PR, partial response*.

*SD, stable disease*.

*PD, progress disease*.

### Efficacy and Adverse Events (AEs) of Nivolumab 3 Months After First Administration

In patients with advanced melanoma, complete response (CR) was seen in 3 patients (6.5%; 95% confidence interval [CI], 0–13.0%), partial response (PR) was seen in 11 patients (23.9%; 95%CI, 0–47.8%), stable disease (SD) was seen in 13 patients (28.3%; 95%CI, 0–56.6%), and progressive disease (PD) was seen in 25 patients (41.3%; 95%CI, 0–82.6%). The objective response rate 3 months after first administration was thus 30.4% (95%CI, 0–60.8%). Tumor responses of individual patients are listed in Table 1. The incidence of AEs was 41.3% (Grade 4: 2.2%, Grade 3: 19.6%, Grade 2: 17.4%, Grade 1: 2.2%) (Table 2).

**Table 2:** Immune-related adverse events in patients with cutaneous melanoma.

	**Adverse events**	**Grade**
1	N.A.	N.A.
2	N.A.	N.A.
3	N.A.	N.A.
4	Bursitis	3
5	Hypophisitis	4
6	Radiation dermatitis	3
7	N.A.	N.A.
8	Thyroid dysfunction	2
9	N.A.	N.A.
10	N.A.	N.A.
11	N.A.	N.A.
12	N.A.	N.A.
13	Thyroid dysfunction	2
14	Thyroid dysfunction	2
15	Psoriasiform dermatitis	3
16	N.A.	N.A.
17	CIDP	3
18	N.A.	N.A.
19	Psoriasiform dermatitis	3
20	N.A.	N.A.
21	N.A.	N.A.
22	N.A.	N.A.
23	N.A.	N.A.
24	N.A.	N.A.
25	Rheumarthritis	3
26	Hypophisitis	2
27	N.A.	N.A.
28	Diarrhea	2
29	Abdominal pain	2
30	Hypophisitis	1
31	N.A.	N.A.
32	Diarrhea	2
33	N.A.	N.A.
34	N.A.	N.A.
35	N.A.	N.A.
36	N.A.	N.A.
37	N.A.	N.A.
38	N.A.	N.A.
39	Diarrhea	2
40	N.A.	N.A.
41	N.A.	N.A.
42	N.A.	N.A.
43	Hypophisitis	3
44	IDDM	3
45	N.A.	N.A.
46	IDDM	3

*CIDP, chronic inflammatory demyelinating polyneuropathy*.

*IDDM, insulin dependent diabetes mellitus*.

### Serum Levels of CXCL5, CXCL10, and CCL22

**Figure 1:**
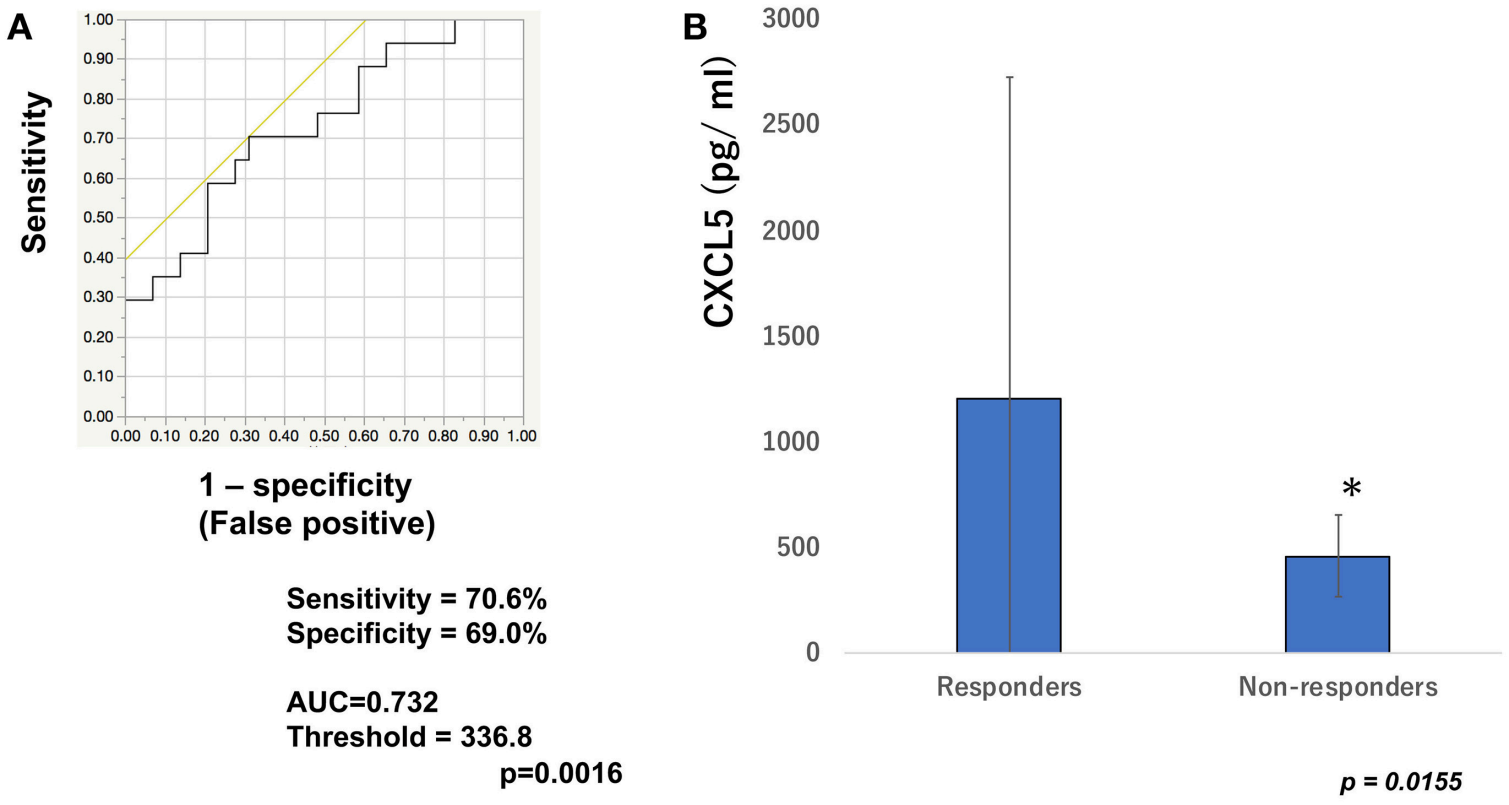
Serum levels of CXCL5 and the ROC curve in melanoma. The ROC curve was used to calculate cut-offs for CXCL5 serum levels and the AUC. Cut-offs were determined to distinguish responders from non-responders using Youden's index **(A)**. Mean serum levels of CXCL5 in responders (*n* = 16) and non-responders (*n* = 30) at day 0 **(B)**. *p < 0.05 (n.s, not significant).

**Figure 2:**
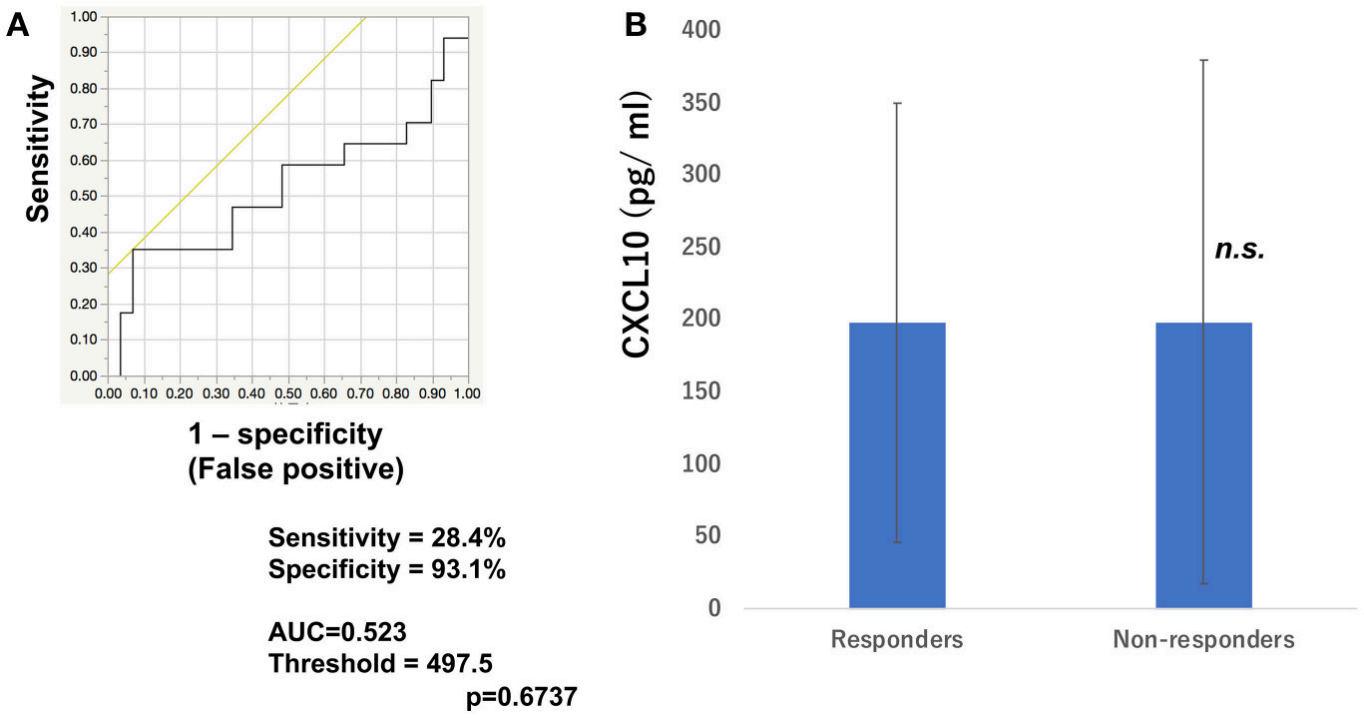
Serum levels of CXCL10 and the ROC curve in melanoma. The ROC curve was used to calculate cut-offs for CXCL10 serum levels and the AUC. Cut-offs were determined to distinguish responders from non-responders using Youden's index **(A)**. Mean serum levels of CXCL10 in responders (*n* = 16) and non-responders (*n* = 30) at day 0 **(B)**. (n.s, not significant).

**Figure 3:**
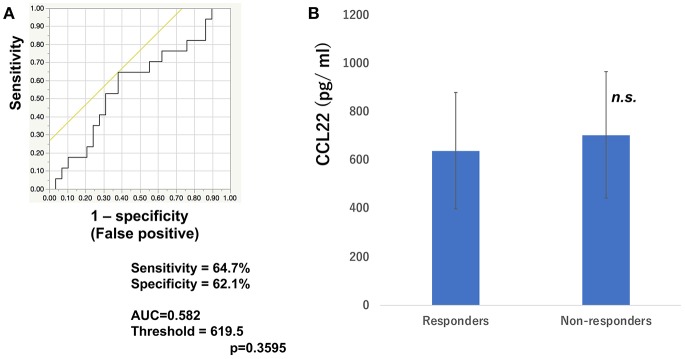
Serum levels of CCL22 and the ROC curve in melanoma. The ROC curve was used to calculate cut-offs for CCL22 serum levels and the AUC. Cut-offs were determined to distinguish responders from non-responders using Youden's index **(A)**. Mean serum levels of CCL22 in responders (*n* = 16) and non-responders (*n* = 30) at day 0 **(B)**. (n.s, not significant).

To determine whether baseline serum levels of CXCL5, CXCL10, and CCL22 may be associated with early response in melanoma patients treated with nivolumab, their levels were evaluated in 46 patients with advanced melanoma treated using nivolumab. Increases in baseline serum CXCL5 and efficacy 3 months after the first administration of nivolumab in each patient are shown in Table 1. The threshold value of CXCL5 at baseline to distinguish responders from non-responders was 497.5 pg/ml. The sensitivity and specificity of the baseline serum CXCL5 in advanced melanoma were 70.6 and 69.0%, respectively (*p* = 0.0016; Figure 1A). High baseline serum levels of CXCL5 were correlated with objective response to nivolumab in patients with advanced melanoma (Figure 1B). On the other hand, there were no significant relationships between serum levels of CXCL10 (Figure 2A) and CCL22 (Figure 3A) and the objective response to nivolumab in patients with advanced melanoma (CXCL10: *p* = 0.674, CCL22: *p* = 0.360). The threshold values of CXCL10 and CCL22 at baseline to distinguish responders from non-responders were 336.8 and 619.5 pg/ml, respectively. There were no significant differences in serum CXCL10 and CCL22 levels in patients with objective response and non-responding patients (Figures 2B, 3B). Baseline serum CXCL5, CXCL10, and CCL22 levels in each patient are shown in Table 1. There were no significant relationships between serum levels of CXCL5 (*p* = 0.0703), CXCL10 (*p* = 0.1748), and CCL22 (*p* = 0.2207) and irAEs in patients with nivolumab-treated advanced melanoma.

## Discussion

As previously reported, increased levels of soluble(s) CD163 at 6 weeks could predict the efficacy of nivolumab monotherapy 2–3 months after its first administration for the treatment of advanced cutaneous melanoma (24). Indeed, the sensitivity and specificity of serum sCD163 for the prediction of efficacy of nivolumab in cutaneous melanoma were 84.6 and 87.0%, respectively (*p* = 0.0030). Moreover, the absolute serum levels of sCD163 (baseline levels of sCD163 compared with day 42) were significantly increased in advanced melanoma patients who developed irAEs (24). This report concludes that the absolute serum levels of sCD163 are useful for the prediction of irAEs in melanoma patients, especially in combination with the absolute value of CXCL5 (25). Since serum sCD163 and CXCL5 are, at least in part, derived from CD163+ TAMs that are activated by periostin (24, 26), and chemokine profiles from TAMs are determined by the stimulation of stromal factors (27), spontaneously produced TAM-related factors could be detected in serum from melanoma patients (17, 25, 27). Notably, CD163+ M2 macrophages could be activated by periostin, leading to the production of characteristic chemokines, such as CXCL5, CXCL10, and CCL22, (28) that are correlated with recruitment of both immunosuppressive cells and immune-reactive anti-tumor cells (25). On the other hand, PD-1 expression is a key factor in maintaining TAMs as M2-polarized, and blockade of PD-1/PD-L1 leads to conversion of TAMs into M1-polarized activated macrophages (29). Since CD163+ TAMs are activated by anti-PD1 antibody (29), the TAM-related chemokines such as sCD163 and CXCL5 are important to evaluate the recruitment of anti-PD1 antibody in the tumor microenvironment.

From the above findings, in this report, we hypothesized that baseline serum levels of TAM-related chemokines, CXCL5, CXCL10, and CCL22, might be correlated with the efficacy of nivolumab in patients with advanced melanomas. To prove this hypothesis, serum levels of CXCL5, CXCL10, and CCL22 were analyzed in 46 cases of advanced melanoma treated with nivolumab. Baseline serum levels of CXCL5 were significantly increased in the response group compared to the non-response group in melanoma. In contrast, no significant differences in baseline serum levels of CXCL10 and CCL22 were seen between the nivolumab response and non-response groups. This discrepancy might be caused by the different sources of CXCL10 and CCL22, such as dendritic cells and endothelial cells that express lower levels of PD1 (29), leading to no effect of anti-PD1 antibody on the production of these chemokines in melanoma patients. Since CXCL5 is also reported as a biomarker for various T helper 17 cell-mediated autoimmune disorders (30–32), the high serum levels of CXCL5 might be correlated with the anti-tumor immune response of anti-PD1 antibody that could also induce autoimmune-like responses such as interstitial pneumonia, autoimmune-like colitis, and hepatitis (33). Taken together, CXCL5 may represent a predictive biomarker for evaluating the efficacy of nivolumab 3 months after its first administration for advanced melanoma. The present study suggested that CXCL5 may be a useful biomarker for the selection of those melanoma patients most likely to benefit from anti-melanoma immunotherapy using nivolumab and ipilimumab combined therapy. Because this was a pilot study, future independent studies with larger patient cohorts are needed to confirm the present findings.

## Data Availability

The raw data supporting the conclusions of this manuscript will be made available by the authors, without undue reservation, to any qualified researcher.

## Ethics Statement

The protocol for this human study was approved by the ethics committee of Tohoku University Graduate School of Medicine, Sendai, Japan (Permit No: 2017-1-064). All methods were performed in accordance with the relevant guidelines and regulations. All patients provided their written, informed consent.

## Author Contributions

TFuj designed the research study. YS, TFuj, KT, CL, and YK gathered and analyzed the ELISA data. TFuj, YK, RA, AO, YF, KY, SM, HU, YY, HH, TFun, YN, RT, HO, NW and AH treated the patients and acquired the clinical data and samples. TFuj wrote the manuscript. TFuj and SA supervised the study.

### Conflict of Interest Statement

The authors declare that the research was conducted in the absence of any commercial or financial relationships that could be construed as a potential conflict of interest.
